# Osteocalcin improves outcome after acute ischemic stroke

**DOI:** 10.18632/aging.102629

**Published:** 2020-01-05

**Authors:** Jiayan Wu, Yunxiao Dou, Wangmi Liu, Yanxin Zhao, Xueyuan Liu

**Affiliations:** 1 Shanghai Tenth Peopleʼs Hospital, Tongji University School of Medicine, Shanghai 200032, China; 2 The Second Affiliated Hospital, College of Medicine, Zhejiang University, Hangzhou 310009, China

**Keywords:** osteocalcin, acute ischemic stroke, NIHSS score, proline hydroxylase 1, pyroptosis

## Abstract

Background: Osteocalcin is related to energy metabolism, memory and the acute stress response, suggesting a relationship between bone and the brain. The need to explore the effect of osteocalcin on acute ischemic stroke is therefore urgent.

Results: Patients with better outcomes had higher serum osteocalcin levels than those whose NIHSS scores did not improve. Multivariable logistic regression analysis showed acceptable performance (area under the curve = 0.766). The effect of osteocalcin on the promotion of neuron survival was confirmed by Cell Counting Kit-8 experiments. In addition, osteocalcin could decrease proline hydroxylase 1 and inhibit the degradation of gasdermin D.

Conclusions: We propose that osteocalcin can improve outcome after acute ischemic stroke in the acute period. By downregulating proline hydroxylase 1, osteocalcin leads glucose metabolism to the pentose phosphate pathway and therefore promotes neuronal survival through inhibiting pyroptosis.

Methods: Demographic data and laboratory results were obtained from patients with ischemic stroke in the acute period for analysis. A receiver operating characteristic curve was used to assess the discrimination of the prediction model. The potential effect of osteocalcin on cerebral ischemia and osteocalcin mechanism were explored in cultured primary rat cerebral cortical neurons treated with oxygen-glucose deprivation and reoxygenation.

## INTRODUCTION

The canonical physiological role of bone has long been considered to be its ability to protect internal organs and facilitate body movement. It is not difficult to view bone as our scaffold because of its structural properties. Therefore, we have lost sight of the roles of bone in other physiological processes. The present characterization of bone as an endocrine organ provides a conceptual framework that may shed light on some unusual aspects of bone [1]. Based on the communication between bone and other organs, bone can influence several physiological processes in an indirect manner mediated by cytokines. For example, osteocalcin, a bone-derived hormone, promotes β-cell proliferation, insulin expression and insulin secretion [2].

Unexpectedly, vascular channels between the brain and the skull bone marrow that provide passage for myeloid cell migration were observed in murine models of stroke and aseptic meningitis [3]. This finding led researchers to search for a novel structure in the bone that can account for the release of bioactive cytokines into the general circulation. Transcortical vessels (TCVs) have been identified in human limb bones [4]. Microscopy has revealed that TCVs cross perpendicularly along the shaft and connect to the periosteal circulation. These results further identify bone as an endocrine organ and provide a possible mechanical explanation for the rapid reaction of bone in the acute stress response [5].

An increasing number of researchers are conducting much research on the relationship between bone and the nerve system. Oury et al. reported that osteocalcin promoted postnatal neurogenesis and memory and also prevented anxiety and depression. In addition, maternal osteocalcin can cross the placenta to promote fetal brain development, such as spatial learning and memory [6]. Recently, osteocalcin was shown to suppress the parasympathetic nervous system in the peripheral organs and enable an acute stress response. This process is initiated by a brain-derived signal in the acute stress response that increases glutamate uptake into osteoblasts [5]. However, there is little in the literature regarding whether osteocalcin can improve outcome in acute ischemic stroke in the acute period. Therefore, the aim of this study was to investigate the effects and mechanism of osteocalcin in acute ischemic stroke.

## RESULTS

### Clinicopathologic characteristics of patients

Eighty-three patients with acute ischemic stroke who met the inclusion criteria entered into this study during the study period. The unimproved group comprised 42 patients, while the improved group comprised 41 patients. The patient characteristics in the cohorts are given in Table 1. Significantly higher osteocalcin and lower fasting blood glucose levels were observed in the improved group compared to the unimproved group. There were no significant differences in age, sex, National Institutes of Health Stroke Scale (NIHSS) score at admission, calcium level, vitamin D level, homocysteine level, or total cholesterol level between the two cohorts.

**Table 1:** Characteristics of the Study Variables.

**Variables**	**Unimproved Group, n=42**	**Improved Group, n=41**	**P**
Age (years)	67.75±10.01	71.67±9.89	0.501
Gender			0.078
Female	8 (19.0%)	16 (39.0%)	
Male	34 (81.0%)	25 (61.0%)	
NIHSS score at admission	3.02±2.15	3.71±2.16	0.143
Osteocalcin (ng/mL)	13.06±5.51	16.94±9.15	0.021
Calcium (mmol/L)	2.22±0.08	2.25±0.08	0.134
Fasting blood glucoses (mmol/L)	7.73±3.02	6.31±2.60	0.025
Vitamin D (ng/ml)	42.31±13.97	41.94±17.92	0.917
Homocysteine (μmol/L)	15.01±0.73	14.24±11.60	0.730
Total cholesterol (mmol/L)	4.68±1.35	4.55±0.92	0.629

### Receiver operating characteristic (ROC) curve analysis

**Figure 1:**
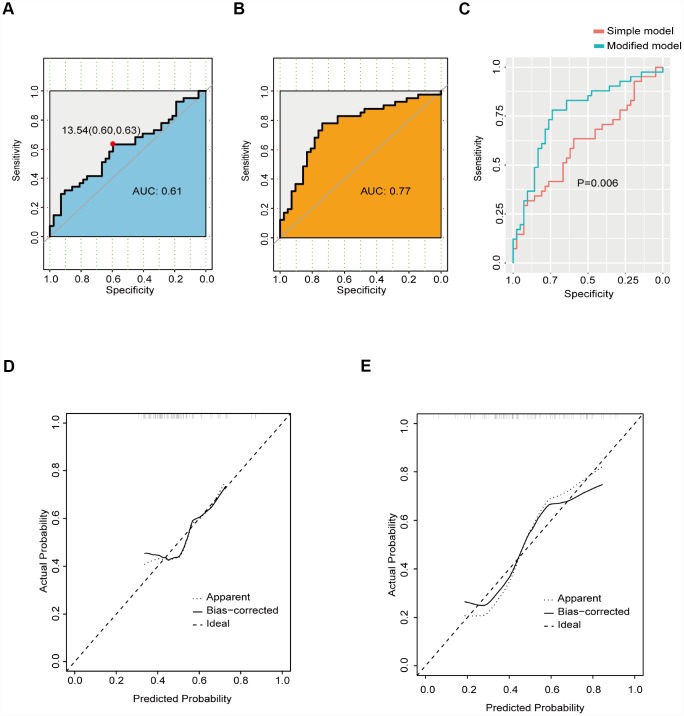
**ROC curve of the prediction model.** (**A**) ROC curve of the simple prediction model based on osteocalcin alone. (**B**) ROC curve of the modified prediction model based on osteocalcin, gender, NIHSS score at admission, calcium level, and fasting blood glucose level. (**C**) Performance comparison between the simple prediction model and the modified prediction model. (**D**) Calibration curve of the simple prediction model. (**E**) Calibration curve of the modified prediction model.

The ROC curve based on osteocalcin is shown in Figure 1A. For osteocalcin, the optimal cutoff value was 13.54 ng/mL, which had a sensitivity of 0.63 and specificity of 0.60. The AUC (area under the curve) for osteocalcin was 0.61. Because a simple prediction model based on osteocalcin alone was not considered acceptable, variables with P values less than 0.15 were selected as candidate factors for a modified prediction model (Figure 1B). The modified prediction model included osteocalcin, sex, NIHSS score at admission, calcium level, and fasting blood glucose level. The AUC for the modified prediction model was 0.77, which was significantly higher than that of the simple prediction model (Figure 1C). The calibration curves for the two models are demonstrated in Figure 1D–1E. The integrated discrimination improvement for the modified prediction model was 0.12 (95% CI: 0.057-0.1864; P<0.001).

### Prognostic nomogram to improve NIHSS score

**Figure 2:**
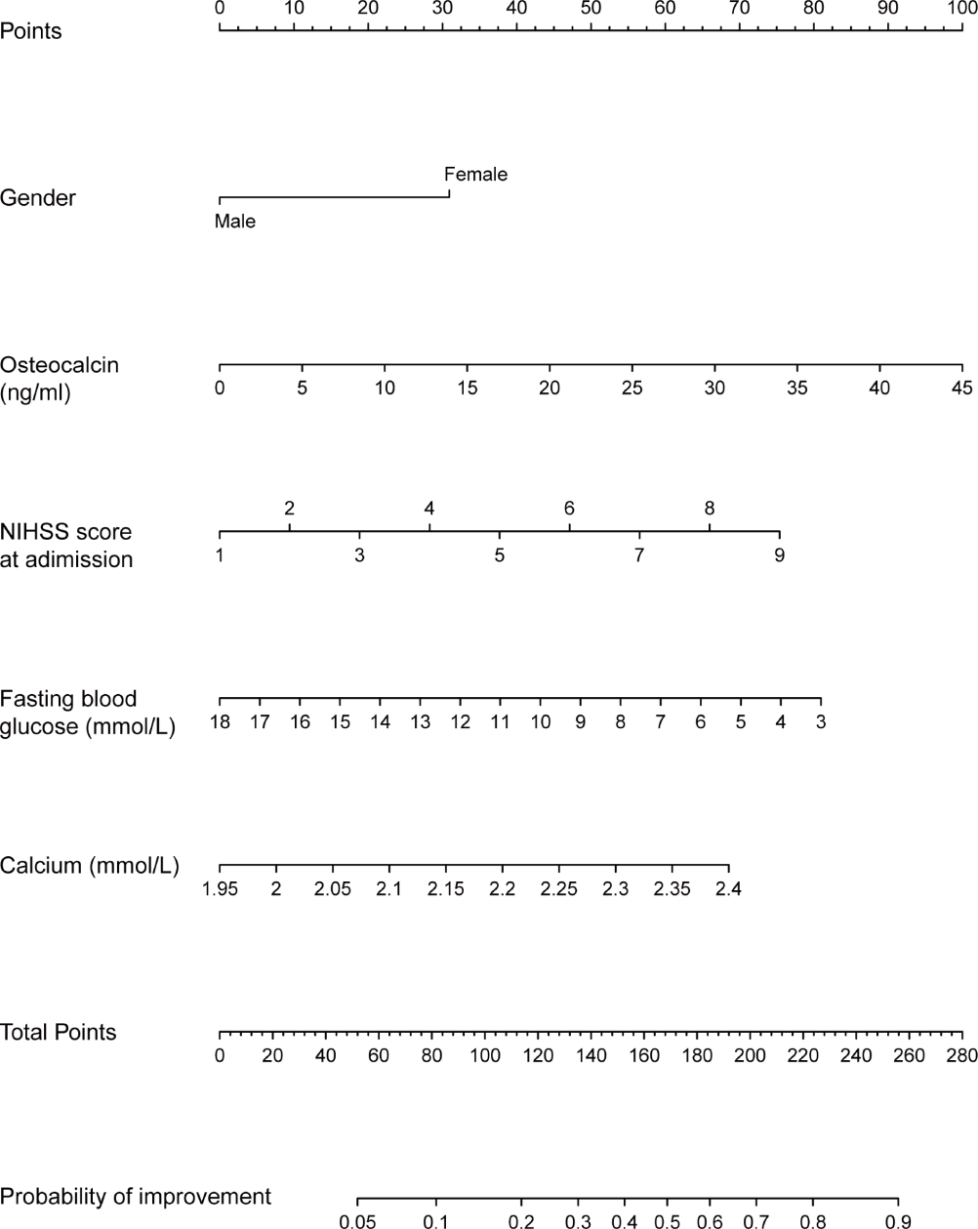
**Nomogram for the probability of NIHSS score improvement.** Instruction for users: locate a patient’s selected variable on the corresponding axis to determine how many points the patient receives for this variable. Add the points awarded for every variable and locate this sum on the total points axis. Draw a line straight down to the Probability of improvement axis to determine the intersection point, which identifies the patient’s probability of NIHSS score improvement.

The prognostic nomogram that integrated all factors in the modified prediction model is shown in Figure 2. Each variable with a given value could be mapped to the points axis. The total points were calculated by adding the points for these variables and are referred to in the total points axis. Then, the probability of NIHSS score improvement in patients with acute ischemic stroke was obtained from the axis named Probability of improvement. For instance, a male patient with low osteocalcin levels is less likely than a woman with high osteocalcin levels to have a good prognosis in acute ischemic stroke.

### Gradient boosting decision tree (GBDT) analysis

**Figure 3:**
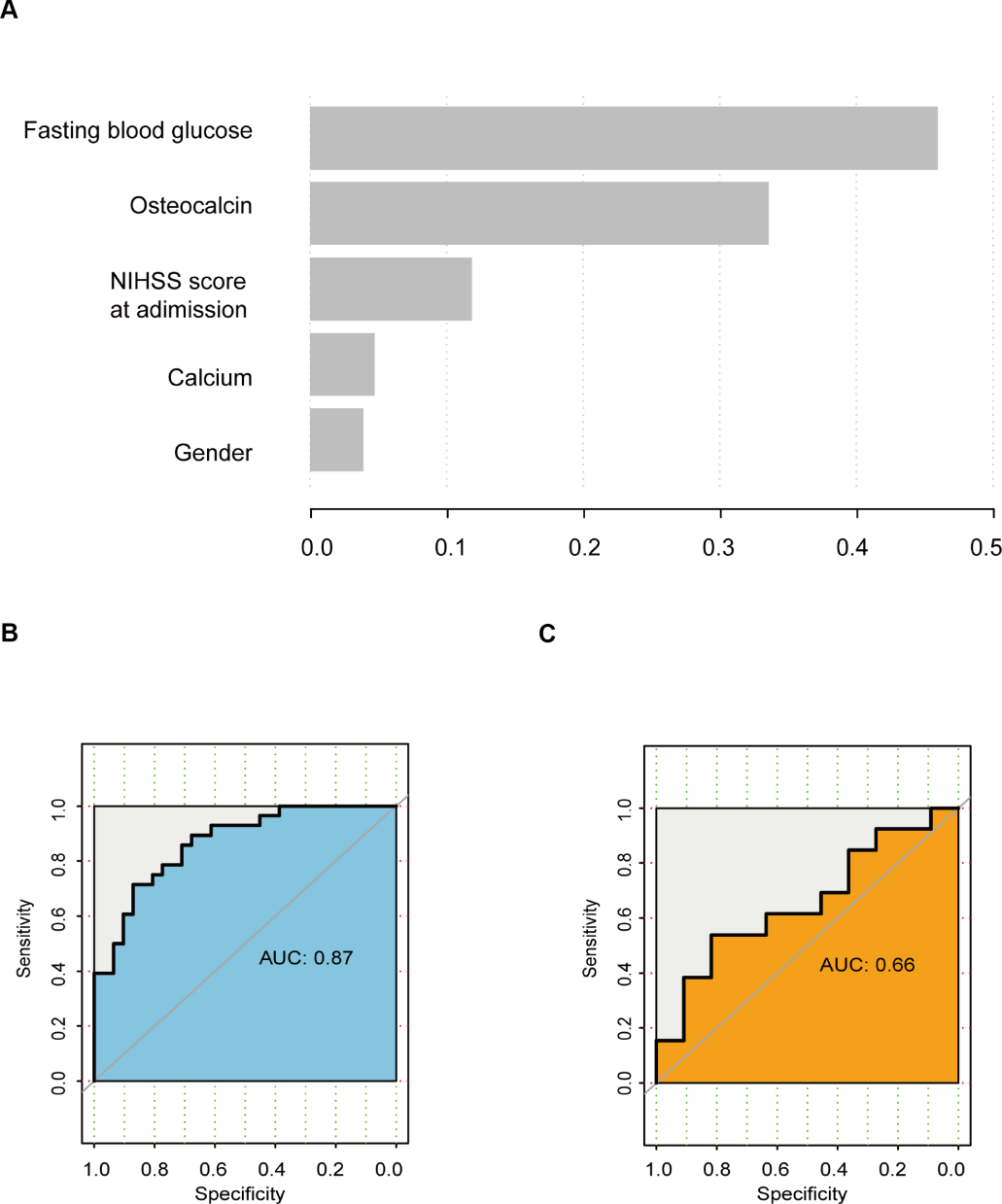
**GBDT analysis.** (**A**) Rank of variables according to their importance. (**B**) ROC curve of the training cohort. (**C**) ROC curve of the validation cohort.

GBDT is a powerful machine learning technique for regression and classification. The variables were ranked according to their importance as follows: fasting blood glucose level, osteocalcin level, NIHSS score at admission, calcium level, and sex (Figure 3A). In the training cohort, the AUC was 0.87 (Figure 3B). However, in the validation cohort, the AUC was 0.66 (Figure 3C).

### CCK-8 assay

**Figure 4:**
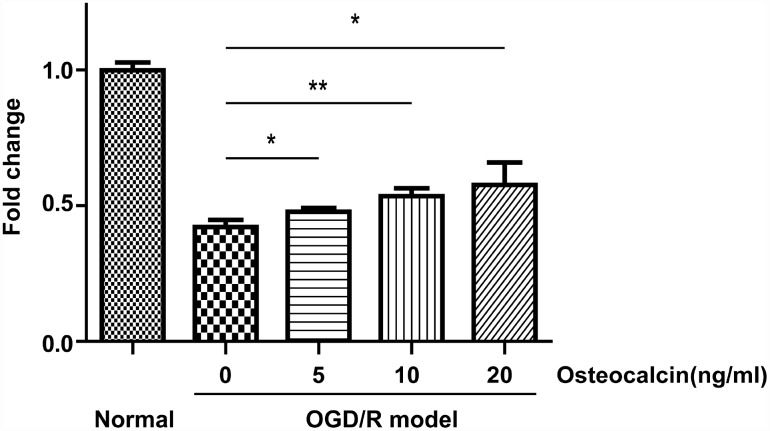
**CCK-8 assay.** Normal group indicates that neurons were incubated in the same buffer containing glucose at 37°C in a regular CO_2_ (5%) incubator. OGD/R had a detrimental effect on the survival of neurons, and this effect could be diminished by the addition of osteocalcin (*, P<0.05; **, P<0.01).

To investigate whether osteocalcin has a protective effect against cerebral ischemia in neurons, we used the CCK-8 assay to evaluate the viability of primary neuronal cells in an oxygen-glucose deprivation and reoxygenation (OGD/R) model. As shown in Figure 4, osteocalcin could promote the survival of neurons when primary neuronal cells were cultured in an extreme environment in a concentration-dependent manner.

### Real-time PCR (RT-PCR) analysis

**Figure 5:**
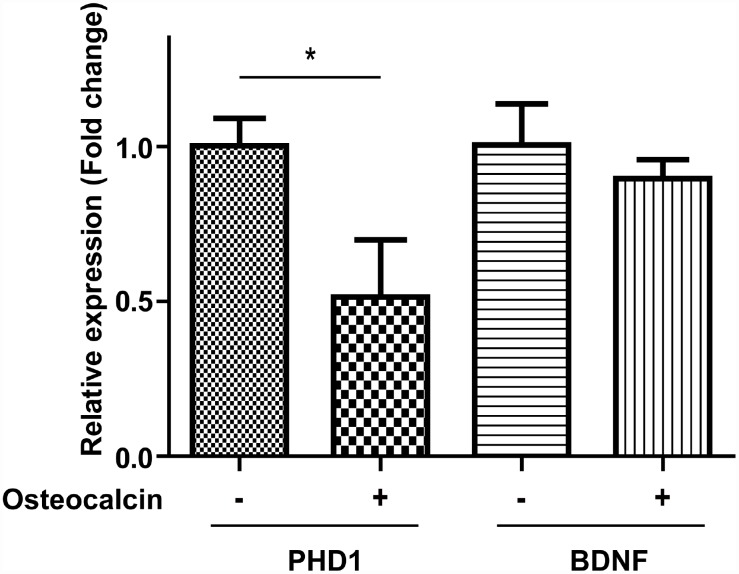
**RT-PCR experiment.** The mRNA expression of PHD1 in the OGD/R model was significantly decreased by the addition of osteocalcin (10 ng/ml) (*, P<0.05).

To explore the possible mechanism for the protective effect of osteocalcin, we used RT-PCR to examine the mRNA expression levels of proline hydroxylase 1 (PHD1) and brain-derived neurotrophic factor (BDNF). PHD1 is related to metabolic reprogramming, and BDNF is a classic neurotrophic factor. There was a significant decline in PHD1 when neurons in the OGD/R model were treated with osteocalcin (P=0.015). On the other hand, the expression level of BDNF was relatively stable with and without osteocalcin (Figure 5).

### Western blotting assay

**Figure 6:**
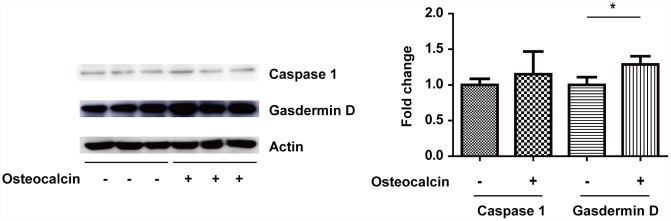
**West blotting experiment.** The total amount of gasdermin D was significantly increased in the presence of osteocalcin (10 ng/ml), which indicates that the degradation of gasdermin D by Caspase 1 was inhibited (*, P<0.05).

The RT-PCR results supported the notion that the inhibition of PHD1 by osteocalcin protects neurons in vitro. To determine the specific kind of neuronal cell death involved in this process, Western blotting was used to detect the expression of gasdermin D and Caspase 1 (Figure 6). Although osteocalcin did not change the expression of Caspase 1, osteocalcin inhibited the degradation of gasdermin D, which is involved in the downstream cellular response to Caspase 1.

## DISCUSSION

This study shows that osteocalcin is beneficial to the outcome of acute ischemic stroke in the acute period. Metabolic reprogramming and decreased pyroptosis account for the protective effect of osteocalcin in an OGD/R model. Although our results do not exclude the possibility that other mechanisms contribute to the function of osteocalcin in cerebral ischemia, these findings support the notion that cytokines from bone are related to many aspects of the nerve system.

Osteocalcin expressed by mature osteoblasts is the most abundant noncollagenous protein found within the bone matrix. As a marker of bone turnover, the circulating level of osteocalcin is used in research and clinical practice to predict fracture risk [7, 8]. Recently, many studies in the literature have investigated the relationship between osteocalcin and stroke [9–12]. However, most of these studies considered osteocalcin to be a bone metabolic index and studied its change after stroke. The lack of knowledge on the effect of osteocalcin on acute ischemic stroke prompted us to assess whether osteocalcin assists in diminishing damage to neurons from acute ischemic stroke. The present clinical data show that a higher osteocalcin level was associated with an increased probability of NIHSS score improvement in patients with acute ischemic stroke. The discriminatory ability of the modified predicton model based on osteocalcin is acceptable. To make the prediction model more user-friendly, we established a nomogram to provide individualized risk assessment. However, the validity of this nomogram needs external validation. Osteocalcin is the second most important factor in GBDT analysis. However, a notable decrease in discriminatory ability was observed in the validation subset, with the AUC decreasing from 0.87 to 0.66. Therefore, we must bear in mind the limited applicability of such selected variables to individualized risk prediction.

The in vitro results indicated that osteocalcin is associated with reduced neuronal death in the OGD/R model and that this effect is likely mediated by PHD1 rather than BDNF. BDNF, a classic neurotrophin, could reduce stroke volume and improve functional outcome in rats with middle cerebral artery occlusion [13]. Furthermore, patients with a high percentage of BDNF-positive Treg cells had a better outcome at 6 months than those with a lower level [14]. However, we found no indication of a significant effect of osteocalcin on BDNF. On the other hand, the production of PHD1 was inversely associated with the presence of osteocalcin. PHD1, which regulates the response to hypoxia, orchestrates an adaptive response to hypoxia and oxidative stress with hypoxia-inducible factors [15]. It was previously confirmed that the loss of PHD1 makes the peripheral organs more tolerant of ischemia by shifting mitochondrial metabolism towards anaerobic glycolysis [16, 17]; however, recent evidence suggests that deletion or inhibition PHD1 shunts more glucose into the antioxidant pentose phosphate pathway while reducing glycolytic flux in neurons [18]. Therefore, the protective effect of osteocalcin against cerebral ischemia is partly attributable to the reprogramming of neuronal metabolism by decreased PHD1.

The area of the brain tissue affected by acute ischemic stroke is divided into the core and penumbra. The core becomes rapidly necrotic due to the deepest ischemia in the central area, whereas cell death occurs in the penumbra later by more diverse mechanisms [19]. Pyroptosis is a form of regulated necrosis mediated by Caspase-1, which cleaves gasdermin D into NH_2_-terminal fragments that oligomerize to form pores in the membrane, causing necrosis [20]. Boc-D-CMK, a Caspase-1 inhibitor, attenuates apoptotic neuronal damage incurred during transient global cerebral ischemia [21]. The results herein showed that osteocalcin did not inhibit Caspase-1, whereas it prevented gasdermin D cleavage. This appeared to be due to metabolic reprogramming in neurons.

Our study has several limitations. First, it is a retrospective study, and we could not control for all potential confounding factors. Second, because the baseline osteocalcin level in patients before stroke is unlikely to be determined, we can reach no strong conclusions regarding release of osteocalcin from bone in stroke as an analogy for osteocalcin release in the acute stress response. Third, the intracellular signaling pathway by which osteocalcin exerts its effects in stroke needs further research.

In summary, the results of the current study demonstrate that osteocalcin attenuates neuronal loss and improves outcome in acute ischemic stroke. These effects are associated with the inhibition of PHD1 and prevention of gasdermin D degradation. Altogether, our findings indicate that osteocalcin can serve as a promising therapeutic strategy in stroke treatment and rehabilitation. However, further study is warranted prior to progressing to the clinical arena.

## MATERIALS AND METHODS

### Patients

From January 2018 to May 2019, a cohort of consecutive patients with acute ischemic stroke was retrospectively studied at Shanghai Tenth People’s Hospital. The inclusion criteria were the following: first onset of ischemic stroke confirmed by MRI within three days; NIHSS scores (with scores ranging from 0 to 42, with a higher score indicating worse symptoms) on admission and on the 7^th^ day available; NIHSS score on admission ranging from 1 to 10; and osteocalcin, vitamin D, total cholesterol, fasting blood glucose, glycosylated hemoglobin, calcium and homocysteine levels detected on admission. The exclusion criteria were as follows: history of previous stroke, history of other disorders of the nervous system, diagnosis 3 days after onset, receipt of thrombolytic therapy, and incomplete information required for analysis.

Patients were categorized according to improvement in NIHSS score on the 7^th^ day compared with that on admission. The improved group was defined as patients exhibiting an improvement of ≥1 point on the 7^th^ day after onset, while the unimproved group was defined as patients exhibiting the same or even a worse score on the 7^th^ day. This study was approved by the Ethics Committee of the Shanghai Tenth People’s Hospital. Informed consent was obtained from participants.

### Primary neuronal cell culture

Specific pathogen-free E18 SD rats were purchased from Shanghai SLAC Laboratory Animal Co., Ltd. (Shanghai, China). The animals were anesthetized with the intraperitoneal administration of chloral hydrate at day 18 of gestation. The bilateral cerebral cortices of fetal rats were removed, minced, dissolved, filtered, and suspended in DMEM (Gibco, USA) containing 10% fetal bovine serum (Gibco, USA). The suspended cells were then seeded on plates coated with poly-lysine (Sigma-Aldrich, USA) and cultured at 37°C in humidified air with 5% CO_2_. After a 4-hour incubation, the medium was replaced with neurobasal medium (Gibco, USA) This study was approved by the institutional Ethics Committee. All procedures were performed carefully to minimize animal suffering.

### OGD/R model

An OGD/R model was established as previously described [22]. The neuronal cells were initially exposed to OGD medium (D-glucose-free Neurobasal A medium, B27, GlutaMax) and then placed in an anaerobic chamber (Plas Labs) containing nitrogen (95%) and CO_2_ (5%) for 2 hours at 37°C. OGD was terminated by adding neurobasal medium, and cell cultures were reintroduced to the regular CO_2_ incubator and incubated at 37°C for 24 hours.

### Cell survival rate analysis

Primary neurons were divided into four groups: a (1) normal group that was incubated in the same buffer containing glucose at 37°C in a regular CO_2_ (5%) incubator during the entire experiment, an (2) OGD/R group that was not treated with osteocalcin, an (3) OGD/R + 5 ng/ml osteocalcin group, an (4) OGD/R + 10 ng/ml osteocalcin group, and an (5) OGD/R + 20 ng/ml osteocalcin group. According to the manufacturer’s instructions, a Cell Counting Kit-8 (Yeason, China) was used to detect neuronal viability. Briefly, primary neurons were collected and seeded in 96-well plates at 2 × 10^4^ cells/well. After the successful construction of the OGD/R model mentioned above, 10 μL of CCK-8 reagent was added to each well and incubated for another 1 hour. Finally, the absorbance at a wavelength of 450 nm was measured with a microplate reader (Bio-Rad Laboratories, Hercules, CA, USA).

### Gene expression analysis by RT-PCR

Primary neurons were divided into two groups: an (1) OGD/R group that was not treated with osteocalcin and an (2) OGD/R + 10 ng/ml osteocalcin group. Total RNA was isolated from neurons by the TRIzol (Invitrogen) method. Then, the total RNA was converted to cDNA with an RT Reagent Kit with gDNA Eraser (TaKaRa, Japan) following the manufacturer's instructions. Subsequently, RT-PCR was performed using an ABI 7500 real-time PCR system (Applied Biosystems, USA). All of the primers were purchased from Sangon Biotech Co., Ltd. (Shanghai, China). The following primers were used in this study: β-actin, sense: 5'- CAACCGCGAGAAGATGACCC -3', antisense: 5'- GAGGCGTACAGGGATAGCAC -3'; PHD1, sense: 5'- GACAAGGCAACTTGGCCTAC -3', antisense: 5'- TCGTCAGACCTCTCGAACCT -3; and BDNF, sense: 5'- CTTTGACCGGTTGCTCATTT -3', antisense: 5'- AACACCTTTCTGTCCCGATG -3. RT-PCR was performed using SYBR Green Mix (TaKaRa, Japan). Relative quantification of the aforementioned genes was determined using the 2^-∆∆^Ct method with β-actin as an endogenous control.

### Western blotting

Primary neurons were divided into two groups: an (1) OGD/R group that was not treated with osteocalcin and an (2) OGD/R + 10 ng/ml osteocalcin group. Neurons were pulverized in lysis buffer with phosphatase and protease inhibitor cocktails (CST, USA). Cell lysates containing equivalent amounts of proteins were subjected to SDS-PAGE using 10% polyacrylamide gels. Afterwards, the proteins were transferred onto polyvinylidene difluoride membranes (Millipore Corporation, USA). The following antibodies were used to blot the membrane in this study: anti-gasdermin D (Abcam, USA), anti-Caspase 1 (Abcam, USA) and anti-Actin (Abcam, USA). Horseradish peroxidase–conjugated anti-IgG was used as the secondary antibody to detect the primary antibody. The bands were detected using chemiluminescence reagents (Thermo Fisher, USA).

### Statistical analysis

For continuous variables, means and standard deviations were calculated, while dichotomous variables were examined as the percent relative to the cohort with and without that variable. The statistical significance of these differences was assessed using the t-test and chi-square test for continuous variables and dichotomous variables, respectively. The significance level was set to P≤0.05.

An ROC curve was used to assess the accuracy of prediction of NIHSS score improvement based on osteocalcin and to determine the cut-off point. Then, variables with P≤0.15 were included in the modified prediction model. A nomogram was formulated based on the modified prediction model by using the rms package in R version 3.6.1. Internal validation of the selected variables was performed using GBDT [23]. We partitioned each sample into training and validation sets using 70%/30% random samples without replacement. The training cohort was used to build models, whereas the validation cohort was used to determine the performance of the model.
